# Non-volatile and volatile compound analyses revealed the effect of oregano essential oil on the flavor characteristics of beef

**DOI:** 10.3389/fnut.2026.1832300

**Published:** 2026-05-07

**Authors:** Pengjia He, Siyu Cheng, Yannan Ma, Qiang Cheng, Zhaomin Lei

**Affiliations:** 1 College of Animal Science and Technology, Gansu Agricultural University, Lanzhou, China; 2 CR NG Fung Co., Ltd, Guangdong, China; 3 College of Life Sciences, Northwest Normal University, Lanzhou, China; 4 Gansu Xukang Food Co., Ltd, Pingliang, China

**Keywords:** amino acids, beef, flavor quality, oregano essential oil, volatile compounds

## Abstract

Growing consumer demand for high-quality beef products has promoted extensive research on natural plant additives to improve meat flavor and quality. Oregano essential oil (OEO), rich in bioactive components such as carvacrol and thymol, has been widely concerned due to its excellent antimicrobial and antioxidant activities, but systematic studies on its regulatory effect on beef flavor characteristics (especially volatile and non-volatile flavor compounds) remain insufficient. This study aimed to explore the effect of dietary OEO supplementation on beef flavor characteristics by comprehensively analyzing non-volatile flavor substances (nutritional composition, amino acid profiles, flavor-related nucleotides) and volatile compound profiles. A total of 27 Pingliang red cattle were randomly divided to three groups: the control group (CON, fed a basal diet), the low OEO supplementation group (LOE, basal diet supplemented with 130 mg/d OEO), and the high OEO supplementation group (HOE, basal diet supplemented with 260 mg/d OEO). Electronic nose and gas chromatography-ion mobility spectrometry (GC-IMS) were used for volatile compound analysis in beef. The results showed that dietary supplementation with HOE significantly increased the content of umami and sweet amino acids, as well as flavor-related nucleotides in beef (*P* < 0.05). A total of 36 volatile compounds were identified by GC-IMS, among which the total contents of aldehydes, alcohols, and ketones were significantly increased in the HOE group. Further analysis confirmed that six volatile compounds, including octanal, pentanal, 3-methylbutanal, 2-butanone, 2-pentanone, and 2-pentyl furan, were the key characteristic flavor contributors. In conclusion, dietary OEO supplementation can positively regulate the flavor profile of beef by enriching non-volatile and volatile flavor compounds, which provides a theoretical basis and practical support for OEO as a natural plant additive to improve beef flavor quality.

## 1 Introduction

Beef is a primary global source of animal protein, renowned for its high-quality protein, unsaturated fatty acids, essential amino acids, and vitamins (1). As consumer demand for beef continues to rise, there is growing interest in products that offer superior nutritional value and eating quality (2, 3). Meat quality is typically assessed through multiple factors, including appearance, quality characteristics, flavor, and nutritional value (4). Among these, flavor is a primary determinant of overall eating quality and plays a critical role in influencing consumer preference and repurchase intent (5). The flavor profile of meat arises from water-soluble small molecules and lipids, which are formed into non-volatile taste components and volatile odor compounds by lipid oxidation, Maillard reaction, and Strecker degradations. The composition and concentration of these substances are essential for evaluating meat flavor quality (5). Therefore, flavor modulation has become a focus in meat science research, given its significant impact on overall product quality and value. Flavor development is closely linked to the animal diet and metabolic processes. Consequently, dietary intervention, particularly through feed additives, has emerged as a practical strategy to enhance the nutritional properties and eating quality of meat in livestock production (6, 7).

Oregano essential oil (OEO), extracted from the natural aromatic plant, is rich in phenolic compounds (e.g., carvacrol and thymol), flavonoids, and terpenoids, which contribute to its antimicrobial, antioxidant, and flavor-enhancing properties (8–10). Dietary OEO supplementation has been shown to beneficially affect meat quality across various species. For instance, in pigs, OEO has been reported to improve the sensory attributes by increasing intramuscular fat content, optimizing n-3 polyunsaturated fatty acid composition, and enhancing antioxidant capacity (11). In broilers, supplementation with 300 mg/kg OEO positively influenced breast meat appearance and sensory quality (12). In ruminants, dietary OEO has been found to inhibit lipid peroxidation and reduce odorous compounds such as malondialdehyde in mutton and beef, thereby contributing to improved meat quality (13, 14).

Despite accumulating evidence supporting the potential of OEO as a feed additive to enhance meat quality (15), its role in modulating beef flavor, particularly through a systematic evaluation of both taste- and aroma-related compounds, remains underexplored. Existing studies have largely focused on meat quality parameters, with limited attention to the integrated relationship between non-volatile and volatile compounds. In this study, we therefore aimed to systematically investigate the effects of dietary OEO supplementation on beef flavor characteristics by evaluating changes in proximate composition, non-volatile compounds (free amino acids and nucleotides), and volatile compounds. The findings are expected to provide a scientific basis for the application of OEO as a dietary strategy to optimize beef flavor and enhance overall meat quality.

## 2 Materials and methods

### 2.1 Animals, diets and experimental design

All experimental procedures involving animals were approved by the Institutional Animal Care and Use Committee of Gansu Agricultural University (Approval NO.: GAU-AST-2020-12). The feeding experiment was conducted at the Pingliang Red Cattle Breeding Center in Jingchuan County (Pingliang, Gansu, China). Twenty-seven healthy Pingliang Red steers (12-month-old, average body weight 270.47 ± 16.27 kg) were randomly allocated into three dietary treatment groups (*n* = 9 per group). The control group (CON) received a basal diet; the other two groups were supplemented with the basal diet + 130 mg/d OEO (LOE) or 260 mg/d OEO (HOE). The OEO doses were initially determined based on our previous studies in sheep (16), converted based on metabolic body weight, and subsequently optimized and adjusted for safety. The basal diets formulated to meet the nutrient requirements for beef cattle [(73), Supplementary Table S1]. The commercial OEO was sourced from Ralco Nutrition Inc. (Marshall, MN, USA) and contained 1.3% essential oil (carvacrol, thymol, terpinene, and p-cymene), carried in inert clinoptilolite. Steers were individually housed in pens. Following a 14-day acclimation period, animals received the experimental diets for 390 days. No deaths or exclusions occurred among the experimental animals during the trial period. Steers were fed twice daily (0700 and 1,500 h) and provided *ad libitum* access to water throughout the entire experimental duration.

### 2.2 Sample collection

At the end of the experimental period, all steers were fasted for 12 h, transported to the commercial abattoir, and then slaughtered according to standardized procedures. The *longissimus thoracis* muscle (approximately 50 g each) was excised from the left carcass between the 12th and 13th ribs. The samples were immediately flash-frozen in liquid nitrogen, and subsequently stored at −80 °C until further analysis of proximate composition, free amino acids, 5′-nucleotides, and volatile compounds.

### 2.3 Proximate composition analysis

The proximate composition analysis of samples was determined using established methods outlined by the AOAC (17). This analysis included measurements of moisture, crude fat, crude protein, and ash content. Moisture content was quantified by drying samples using a convection oven at 105 °C for 12 h. Crude protein content was analyzed using the Kjeldahl method, where nitrogen content was determined and then multiplied conversion factor of 6.25 to calculate protein content. Crude fat was performed using a Soxhlet extraction method. Ash content was determined by incinerating sample in a muffle furnace at 550 °C.

### 2.4 Free amino acids analysis

Amino acids were quantified using an optimized protocol adapted from (18). Briefly, a 1.0 g muscle samples were homogenized in 15 ml of 5% trichloroacetic acid for 12 h static extraction at 4 °C. The homogenate was centrifuged at 12,000 × g for 15 min at 4 °C. The supernatant was adjusted to pH 2.0 with 6M NaOH under ice-bath stabilization and diluted with 0.1M lithium citrate buffer. Prior to analysis, solutions were filtered through 0.22 μm nylon syringe filters and loaded onto an amino acid analyzer (L-8900, Hitachi, Tokyo, Japan).

### 2.5 5′ -nucleotides quantitative analysis

To analyze 5′-nucleotides, the sample was homogenized with 20 ml of 5% perchloric acid at 15,000 × g for 90 s. The homogenate was centrifuged at 10,000 × g for 10 min at 4 °C, with the resultant re-extracted through identical homogenization/centrifugation cycles. The combined supernatants were adjusted to pH 6.4 using 6 M NaOH, then diluted to 100 mL with ultra-pure water. The solution was filtered through 0.22 μm filters membranes prior to injection and then analyzed by high-performance liquid chromatography (Waters, Milford, MA, USA). The chromatographic conditions were as follows: a C18 column (5 μm, 4.6 × 250 mm; Waters), column temperature at 25 °C. The mobile phase comprised (A) 20 mM potassium phosphate (pH 5.5) and (B) methanol. The flow rate was set at 1.0 ml/min, and the injection volume was 10 μl. Detection wavelength was set at 254 nm. For quantitative analysis, mixed standard solution containing all target nucleotides was prepared to establish the standard calibration curves. Each nucleotide was quantified based on its peak area using the corresponding calibration curve.

### 2.6 Electronic nose analysis

Volatile compound profiling was conducted using an electronic nose system (PEN3, AIRSENSE, Germany) equipped with 10 metal oxide semiconductor sensors. 3.0 g homogenized sample was sealed into a 20 ml headspace vial, and then incubated at 45 °C for 30 min to allow the equilibration of volatile compounds. Subsequently, the headspace gas was automatically injected into the sensor array system detection. The assay time and washing time were 90 s and 60 s, respectively.

### 2.7 Volatile compounds profiling by GC-IMS

Volatile compounds were analyzed using a GC-IMS system (FlavourSpec, G.A.S., Germany) equipped with a 15 m × 0.53 mm × 1 μm MXT-5 capillary column (Restek Corporation, USA) and an autosampler (CTC Analytics AG, Zwingen, Switzerland) with a headspace sampling unit and a gastight syringe (Gerstel GmbH, Mühlheim, Germany). Briefly, 3.0 g samples were thawed at 4 °C for 24 h, and then homogenized in a 20 ml headspace vial (HM-2075G, HAMAG, Ningbo, China). The sealed vial was equilibrated at 60 °C for 15 min. Splitless injection of 500 μl headspace gas was performed via a heated gastight syringe under the following conditions: column temperature at 60 °C, drift tube at 45 °C (150 ml/min N_2_ drift gas), and carrier gas programmed from 2 ml/min (0–2 min) to 100 ml/min (2–18 min). Data acquisition utilized the National Institute of Standards and Technology (NIST) database and linear retention index (RI) calibration. The quantification was employed using the normalized peak area method.

### 2.8 Statistical analysis

Statistical analyses were performed using one-way ANOVA with Tukey's post hoc comparisons in SPSS 26.0 (IBM Corp., Armonk, USA). All data were expressed as means ± SEM, with statistical significance set at *P* < 0.05. Principal components analysis (PCA) of electronic nose data and orthogonal partial least squares discriminant analysis (OPLS-DA) of volatile profiles were performed using *R* software (v3.5.1). Graphs were created using Origin 2021 (OriginLab, MA, USA).

## 3 Results

### 3.1. Proximate composition

The effects of dietary OEO supplementation on the proximate composition of beef were presented in Table 1. No significant differences were observed in moisture, crude fat, crude protein, or ash content among the groups (*P* > 0.05). However, a numerical increasing trend in crude fat content was detected in the HOE group (P = 0.065), which exhibited a 41.2% elevation (4.01% vs. 2.84%) compared to the CON group.

**Table 1:** Proximate composition of beef from cattle fed diets with OEO.

Content (g/100g)	CON	LOE	HOE
Moisture	72.62 ± 0.40	72.19 ± 0.40	72.03 ± 0.69
Crude protein	20.44 ± 0.37	20.41 ± 0.61	21.16 ± 0.47
Crude fat	2.84 ± 0.17	3.51 ± 0.48	4.01 ± 0.20
Ash	1.17 ± 0.01	1.15 ± 0.02	1.12 ± 0.02

Data are presented as means ± SEM (*n* = 9). Different superscript letters within a row indicate significant differences (*P* < 0.05). CON, control diet; LOE, diet supplemented with 130 mg/d OEO diet; HOE, diet supplemented with 260 mg/d OEO diet.

### 3.2 Free amino acid analysis

**Figure 1:**
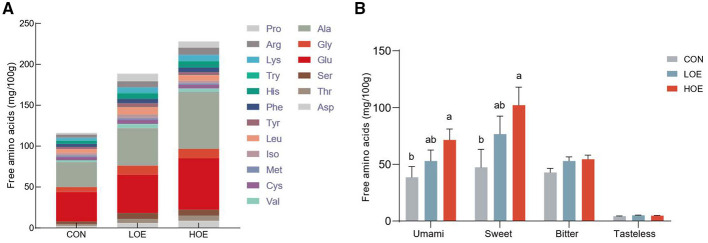
Free amino acid profiles of beef from cattle fed diets with OEO **(A)** composition and content of amino acids; **(B)** flavor amino acids content. Umami amino acids = Asp+ Glu; Sweet amino acids = Ala + Gly + Pro + Ser + Thr; Bitter amino acids = Arg + His + Iso + Leu +Met + Val+ Phe + Tyr + Lys +Try; Tasteless amino acids = Cys. Data are presented as means ± SEM (*n* = 9). Different letters in bar indicated significant differences (*P* < 0.05). CON, Control diet; LOE, diet supplemented with 130 mg/d OEO diet; HOE, diet supplemented with 260 mg/d OEO diet.

Amino acid profiles in beef from different dietary treatments was presented in Figure 1A. 18 amino acids were identified across all groups. OEO supplementation significantly elevated total free amino acid content compared to the CON group, particularly in the HOE group (*P* < 0.05). Amino acids were categorized based on their taste characteristics, including umami, sweet, bitter, or tasteless (19). As shown in Figure 1B, the HOE group exhibited an increase in umami amino acids (Asp and Glu) and sweet amino acids (Gly, Ala, Thr, and Ser) compared to the CON group (*P* < 0.05). No significant differences were observed for bitter and tasteless amino acids across all groups (*P* > 0.05).

### 3.3 5′-Nucleotide analysis

Nucleotide content in beef following the OEO supplementation were shown in Table 2. Dietary OEO significantly elevated adenosine diphosphate (ADP) content compared to the CON group (*P* < 0.05), while no significant differences were observed for adenosine triphosphate (ATP), adenosine monophosphate (AMP), inosine monophosphate (IMP), or guanosine monophosphate (GMP) (*P* > 0.05). IMP emerged as the predominant flavor nucleotide, constituting highest content of 5′-nucleotides across all groups. Notably, the HOE group showed numerically elevated IMP (11.38%) and GMP (31.25%) levels compared to the CON group.

**Table 2:** Nucleotide content of beef from cattle fed diets with OEO.

Content (mg/g)	CON	LOE	HOE
ATP	3.15 ± 0.08	3.44 ± 0.36	3.71 ± 0.14
ADP	0.63 ± 0.02^b^	0.73 ± 0.02^a^	0.80 ± 0.06^a^
AMP	0.43 ± 0.05	0.32 ± 0.08	0.38 ± 0.06
IMP	2.81 ± 0.11	2.98 ± 0.22	3.13 ± 0.17
GMP	0.16 ± 0.02	0.20 ± 0.04	0.21 ± 0.03

ATP, adenosine triphosphate; ADP, adenosine diphosphate; AMP, Adenosine monophosphate; IMP, inosine monophosphate; GMP, guanosine monophosphate.

Data are presented as means ± SEM (*n* = 9). Different superscript letters within a row indicate significant differences (*P* < 0.05). CON, control diet; LOE, diet supplemented with 130 mg/d OEO diet; HOE, diet supplemented with 260 mg/d OEO diet.

### 3.4 Electronic nose analysis

**Figure 2:**
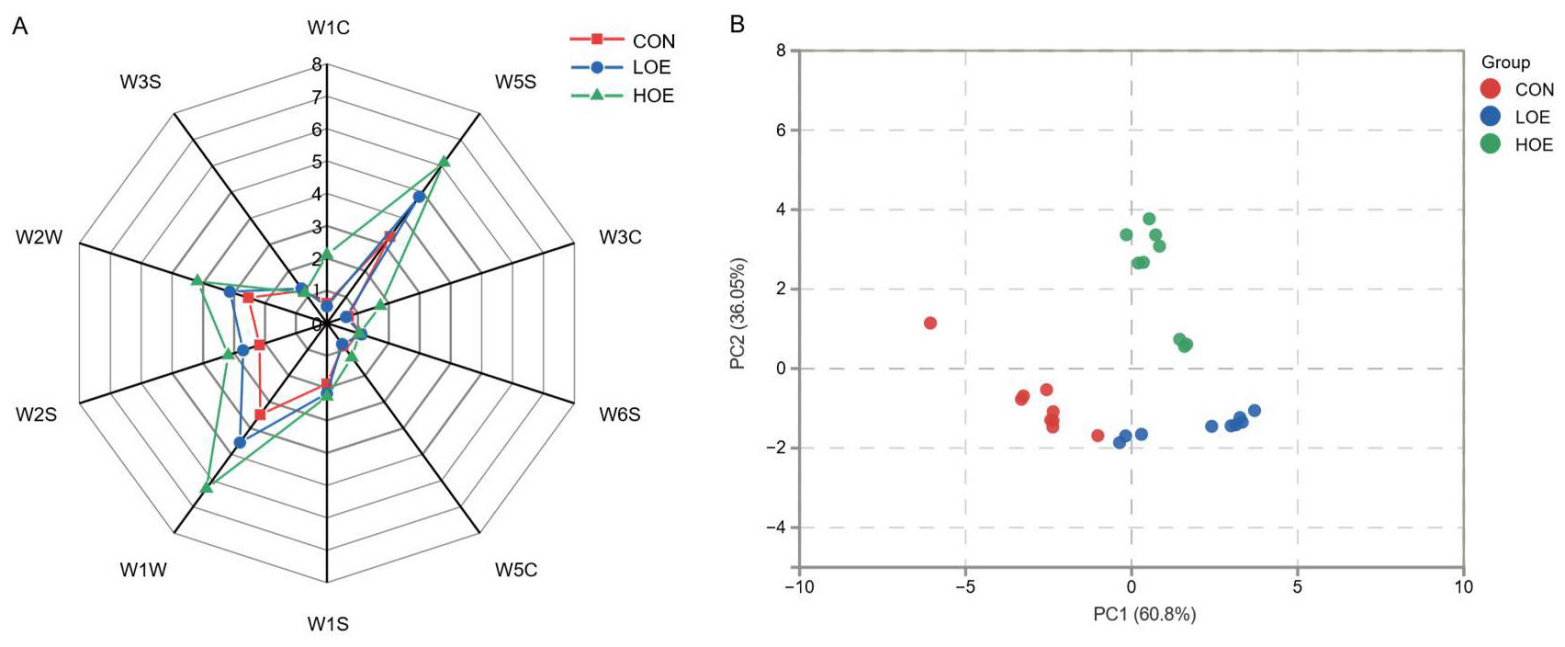
Electronic nose analysis of beef volatiles **(A)** radar chart of sensor responses; **(B)** PCA score plot. CON, Control diet; LOE, diet supplemented with 130 mg/d OEO diet; HOE, diet supplemented with 260 mg/d OEO diet.

The analysis of radar chart revealed distinct differences in the overall odor characteristics among the groups based on the electronic nose sensor response patterns (Figure 2A). Compared to the CON group, the OEO groups exhibited higher response intensities in sensors W1W (sulfides), W5S (nitrogen oxides), and W2S (aromatic components/organic sulfides). Principal Component Analysis (PCA) further confirmed a clear separation among the groups without overlap (Figure 2B), indicating distinct odor characteristics. The relative distance of the E-nose reflected the difference in the overall odor profiles between samples. The larger relative distances were found between HOE groups and CON and LOE groups, indicating more pronounced differences in their overall odor profiles. To further characterize the specific volatile compounds underlying the distinct odor profiles revealed by the electronic nose, the volatile profiles were subsequently analyzed using GC-IMS.

### 3.5 Volatile compound profiles

**Figure 3:**
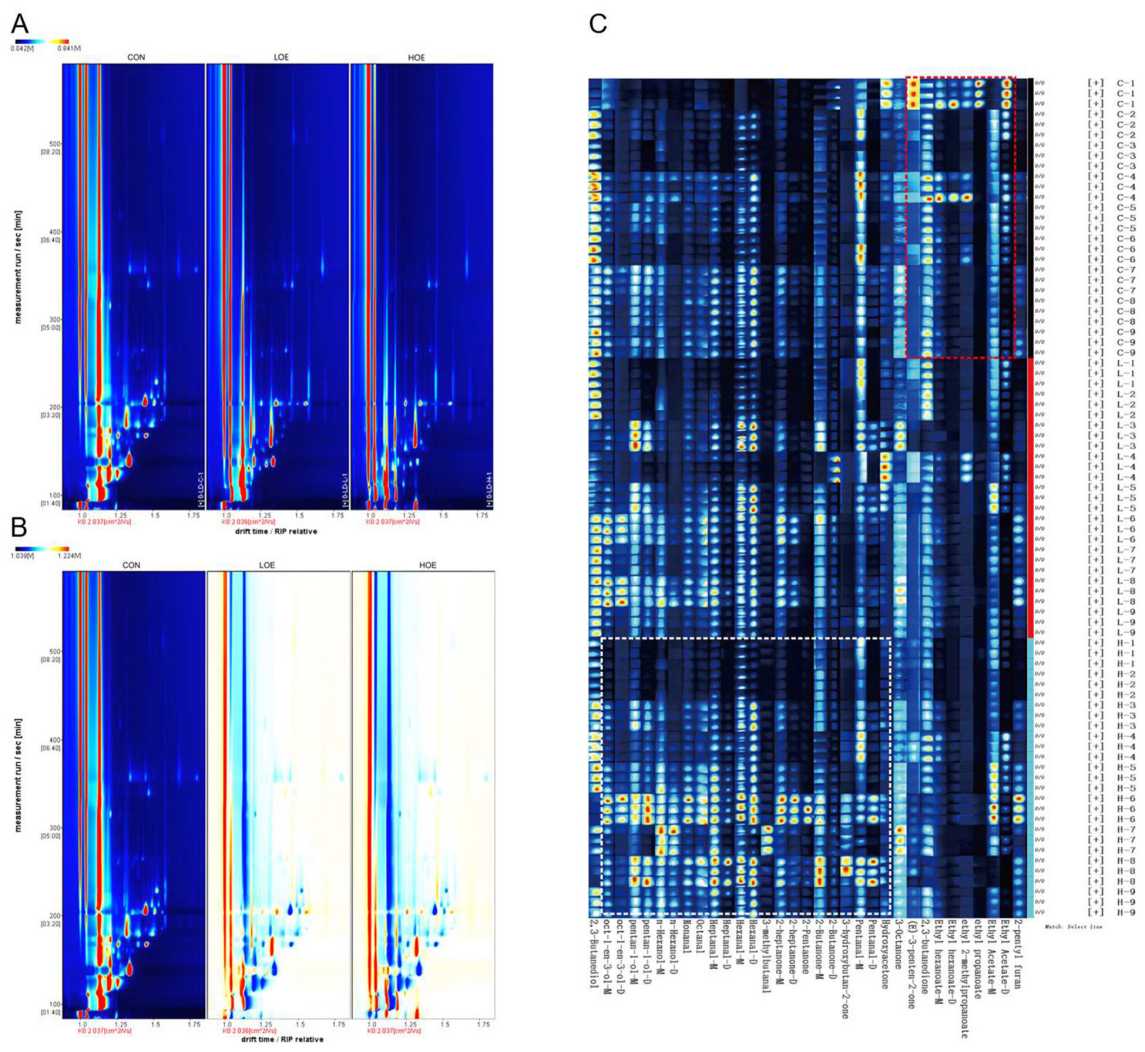
Identification of key volatile compounds in beef by GC-IMS **(A)** two-dimensional topographic plots; **(B)** comparison topographic plots; **(C)** fingerprint of volatile compounds. Color intensity represented the content of volatile compound, and the brighter the color is, the higher the content. CON, Control diet; LOE, diet supplemented with 130 mg/d OEO diet; HOE, diet supplemented with 260 mg/d OEO diet.

Volatile compounds profiles of beef from the CON and OEO groups were characterized using GC-IMS. Distinct differences in volatile compounds were observed across dietary treatments, as illustrated in the two-dimensional spectral plot (Figure 3A). Comparative analysis using the CON group as a reference (Figure 3B) revealed notable differences of volatile compounds in OEO groups, with red intensity gradients indicating elevated compound contents and blue gradients denoting reduced contents.

Building on these spectral differences, fingerprint analysis was employed to resolve the compositional basis of OEO-induced volatile profile alterations. Volatile compound fingerprints derived from GC-IMS signal peak clustering revealed differences in beef across the groups (Figure 3C). Chromatographic signatures demonstrated concentration-dependent color gradients, with darker hues indicating higher volatile compounds contents. Analysis of 50 detected signal peaks identified 36 volatile compounds across five chemical classes, including 9 aldehydes, 10 alcohols, 10 ketones, 6 esters, and 1 furan (Figure 3C, Table 3). Of these, OEO supplementation significantly modulated 19 compounds.

**Table 3:** Volatile compounds of beef from cattle fed diets with OEO.

Compounds	LRI	Relative content/C			Method of identification^1^	Odor description^2^
		CON	LOE	HOE		
Aldehydes						
Nonanal	1,104	1.69 ± 0.11^b^	2.22 ± 0.08^a^	2.42 ± 0.14^a^	MS+LRI	Fatty, green, fruity
Octanal	1,007	0.67 ± 0.33^b^	1.10 ± 0.11^a^	1.34 ± 0.10^a^	MS+LRI	Fatty, green, fruity
Heptanal-M	906	2.23 ± 0.20^b^	2.87 ± 0.19^ab^	3.54 ± 0.22^a^	MS+LRI	Fatty, citrus
Heptanal-D	906	0.91 ± 0.13^b^	0.90 ± 0.09^b^	1.82 ± 0.31^a^	MS+LRI	Fatty, citrus
Hexanal-M	802	4.03 ± 0.32^b^	5.38 ± 0.43^a^	5.46 ± 0.23^a^	MS+LRI	Green, grassy
Hexanal-D	802	6.98 ± 0.67	8.66 ± 0.92	8.25 ± 0.76	MS+LRI	Green, grassy
Pentanal-M	700	3.16 ± 0.28	2.73 ± 0.30	2.47 ± 0.23	MS+LRI	Green, pungent, floral
Pentanal-D	700	0.26 ± 0.04^b^	0.53 ± 0.10^ab^	0.68 ± 0.16^a^	MS+LRI	Green, pungent, floral
3-Methylbutanal	656	0.23 ± 0.01^c^	0.37 ± 0.02^b^	0.99 ± 0.28^a^	MS+LRI	Chocolate, nutty, caramel
Alcohols						
Oct-1-en-3-ol-M	983	1.99 ± 0.16	2.53 ± 0.24	2.22 ± 0.18	MS+LRI	Mushroom
Oct-1-en-3-ol-D	983	0.22 ± 0.01	0.32 ± 0.04	0.25 ± 0.03	MS+LRI	Mushroom
3-Methylbutan-1-ol	730	0.23 ± 0.02	0.25 ± 0.02	0.23 ± 0.02	MS+LRI	Malty, fruity, alcohol
Pentan-1-ol-M	766	1.81 ± 0.21^b^	2.80 ± 0.36^a^	2.61 ± 0.20^ab^	MS+LRI	Balsamic
Pentan-1-ol-D	766	1.78 ± 0.23	2.25 ± 0.33	2.44 ± 0.36	MS+LRI	Balsamic
n-Hexanol-M	884	1.41 ± 0.10^b^	1.56 ± 0.12^b^	2.12 ± 0.17^a^	MS+LRI	Grassy, fatty, fruity
n-Hexanol-D	884	0.13 ± 0.02^b^	0.13 ± 0.02^b^	0.34 ± 0.05^a^	MS+LRI	Grassy, fatty, fruity
1-Propanol	551	1.89 ± 0.53	3.20 ± 1.26	1.14 ± 0.14	MS+LRI	Alcohol, pungent
2,3-Butanediol	786	4.16 ± 0.30^a^	3.37 ± 0.29^ab^	2.83 ± 0.26^b^	MS+LRI	fruity, creamy, buttery
1-Penten-3-ol	675	0.17 ± 0.03	0.13 ± 0.01	0.14 ± 0.01	MS+LRI	Green, fruity
Ketones						
3-Octanone	998	0.18 ± 0.01	0.21 ± 0.01	0.21 ± 0.02	MS+LRI	Earthy, mushroom
Hydroxyacetone	630	0.37 ± 0.05	0.47 ± 0.07	0.32 ± 0.02	MS	
2-Heptanone-M	898	1.24 ± 0.10^b^	1.83 ± 0.16^a^	1.75 ± 0.13^a^	MS+LRI	Fruity, blue cheese
2-Heptanone-D	898	0.28 ± 0.04^b^	0.50 ± 0.10^ab^	0.72 ± 0.13^a^	MS+LRI	Fruity, blue cheese
2-Pentanone	682	0.96 ± 0.04^c^	1.30 ± 0.13^b^	2.56 ± 0.49^a^	MS+LRI	
3-Penten-2-one	710	0.16 ± 0.02	0.16 ± 0.01	0.18 ± 0.01	MS+LRI	Almond, buttery
3-Hydroxybutan-2-one	739	0.71 ± 0.06^b^	0.80 ± 0.10^b^	2.06 ± 0.35^a^	MS+LRI	
2-Butanone-M	604	0.84 ± 0.07^b^	1.32 ± 0.13^a^	1.67 ± 0.15^a^	MS+LRI	Fruity, creamy
2-Butanone-D	597	2.68 ± 0.16^b^	4.02 ± 0.60^a^	2.91 ± 0.14^ab^	MS+LRI	Fruity, creamy
2,3-Butanedione	614	1.77 ± 0.33	1.48 ± 0.12	1.51 ± 0.12	MS+LRI	Caramel, buttery, creamy
Esters						
Ethyl hexanoate-M	1003	1.02 ± 0.54^a^	0.64 ± 0.04^b^	0.63 ± 0.06^b^	MS+LRI	Strong, fruity, winey
Ethyl hexanoate-D	1003	0.42 ± 0.11^a^	0.20 ± 0.01^b^	0.23 ± 0.01^b^	MS+LRI	Strong, fruity, winey
Ethyl 2-Methyl propanoate	756	0.15 ± 0.03^a^	0.11 ± 0.03^ab^	0.05 ± 0.00^b^	ms+lri	Fruity
Ethyl propanoate	709	0.58 ± 0.22	0.22 ± 0.02	0.19 ± 0.02	MS+LRI	Strong, fruity, winey
Ethyl acetate-D	615	10.21 ± 1.81^a^	4.75± 1.08^b^	4.31 ± 0.94^b^	MS+LRI	Sharp, winey
Ethyl acetate-M	615	1.54 ± 0.12	1.46 ± 0.13	1.54 ± 0.15	MS+LRI	Sharp, winey
Furan						
2-Pentyl furan	994	0.35 ± 0.04^b^	0.53 ± 0.06^ab^	0.72 ± 0.08^a^	MS+LRI	Green, fatty, fruity

Data are presented as means ± SEM (*n* = 9). Different superscript letters within a row indicate significant differences (*P* < 0.05). M, monomer. D, dimer. CON, control diet; LOE, diet supplemented with 130 mg/d OEO diet; HOE, diet supplemented with 260 mg/d OEO diet.

^1^MS+LRI, mass spectrum and LRI agree with those of authentic compounds; ms+lri, mass spectrum and LRI in agreement with the literature; MS, mass spectrum agrees with spectrum in the NIST Database.

^2^odor descriptions of volatile compounds were mainly obtained from the literature and online database (34–36), (http://www.flavornet.org).

Obviously, among these volatile compounds influenced by OEO, hexanal emerged as the most abundant volatile compound across all dietary groups, representing approximately 20% of total volatile compounds in beef. OEO supplementation significantly increased the contents of nonanal, octanal, hexanal-M, and 3-methylbutanal compared to the CON group (*P* < 0.05), with the HOE group specifically increasing the contents of heptanal and pentanal-D (*P* < 0.05). Among the detected alcohols, pentan-1-ol-M and n-hexanol exhibited the higher contents in the OEO groups, while 2,3-butanediol demonstrated a significant decrease (*P* < 0.05). Regarding ketones, HOE supplementation significantly increased the contents of 2-heptanone-M, 2-heptanone-D, 3-hydroxybutan-2-one, and 2-butanone-M compared to the CON group (*P* < 0.05), while LOE increased 2-heptanone-M, 3-hydroxybutan-2-one, 2-butanone-M, 2-butanone-D (*P* < 0.05). Notably, OEO supplementation significantly reduced esters contents including ethyl hexanoate-M, ethyl hexanoate-D, and ethyl acetate-D compared to the CON group. Additionally, HOE decreased ethyl 2-methyl propanoate (*P* < 0.05). While furan was detected in minor levels across all groups, a statistically significant difference was observed between the CON and HOE groups (*P* < 0.05).

**Figure 4:**
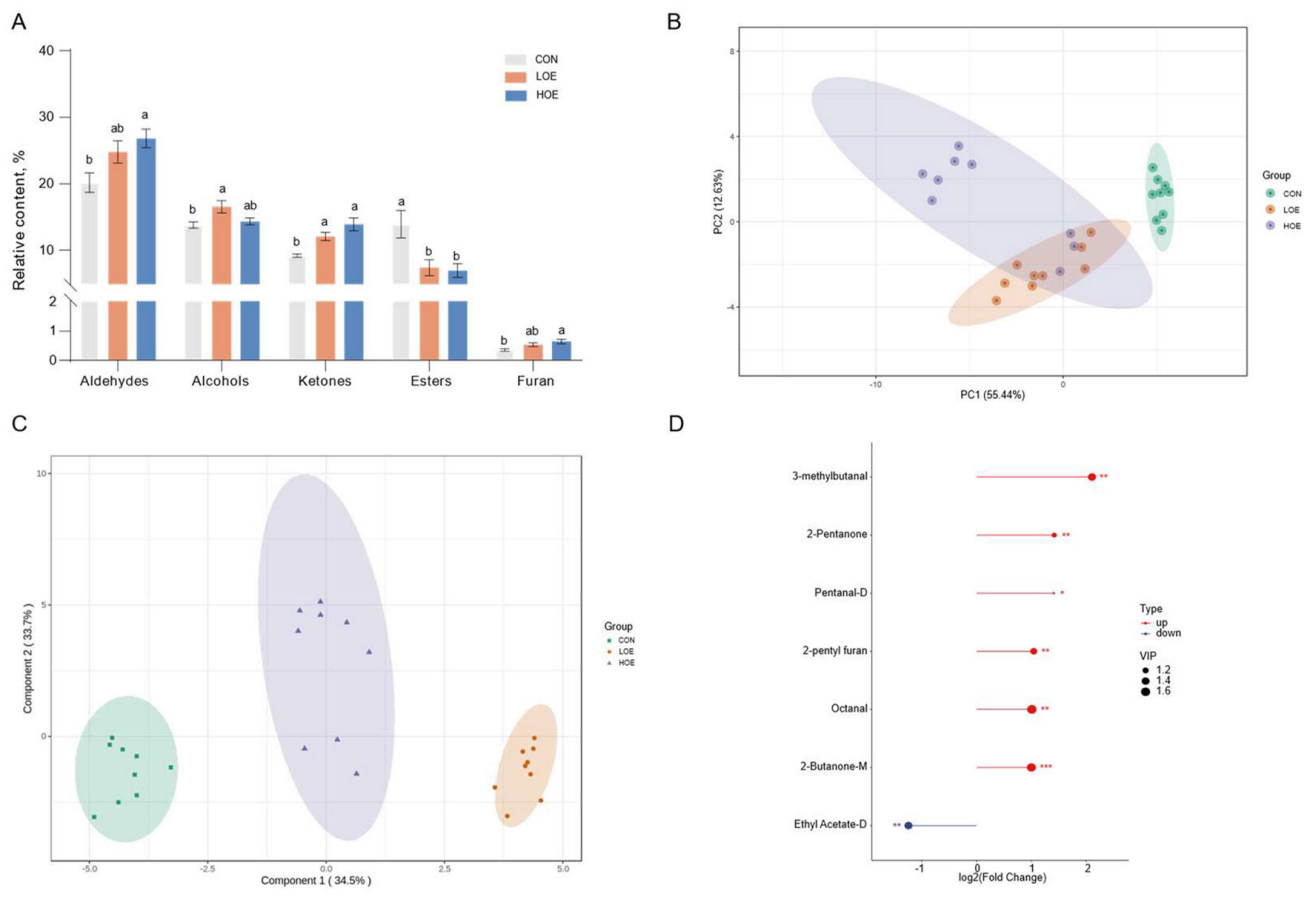
Multivariate analysis of volatile compounds **(A)** the relative percentage of volatile class; **(B)** PCA score plot; **(C)** OPLS-DA score plot; **(D)** FC and VIP scores of volatile compounds. Data are presented as means ± SEM (*n* = 9). Different letters in bar indicate significant differences (*P* < 0.05). CON, Control diet; LOE, diet supplemented with 130 mg/d OEO diet; HOE, diet supplemented with 260 mg/d OEO diet.

The analysis of the total relative percentages revealed distinct compositional shifts in major volatile compound categories (Figure 4A). Aldehydes were the most abundant volatiles identified in beef, followed by alcohols, ketones, esters, and furan. Notably, the HOE group exhibited significantly higher total contents of aldehydes, alcohols, ketones, and furan compared to the CON group (*P* < 0.05). Conversely, the total ester content was significantly reduced in the HOE group (*P* < 0.05).

### 3.6. Multivariate analysis of volatile compounds

Multivariate statistical analysis to identify the key flavor differences contributors across dietary groups. As illustrated in Figure 4B, PCA analysis demonstrated effective differentiation between the CON and OEO groups, reflecting divergent volatile profiles. OPLS-DA model further revealed distinct clustering among the three dietary treatments, confirming significant intergroup volatile composition differences (Figure 4C). Based on a combined criterion of OPLS-DA-derived variable importance in projection (VIP > 1), *P* < 0.05, and |log2FC| ≥ 1, a total of 7 key differential compounds were identified. Among them, six compounds, including 3-methylbutanal, pentanal-D, octanal, 2-butanone-M, 2-pentanone, and 2-pentyl furan were found to be significantly elevated in the HOE group. These markers collectively define the flavor signature differentiating HOE and CON groups (Figure 4D).

### 3.7 Correlation analysis

**Figure 5:**
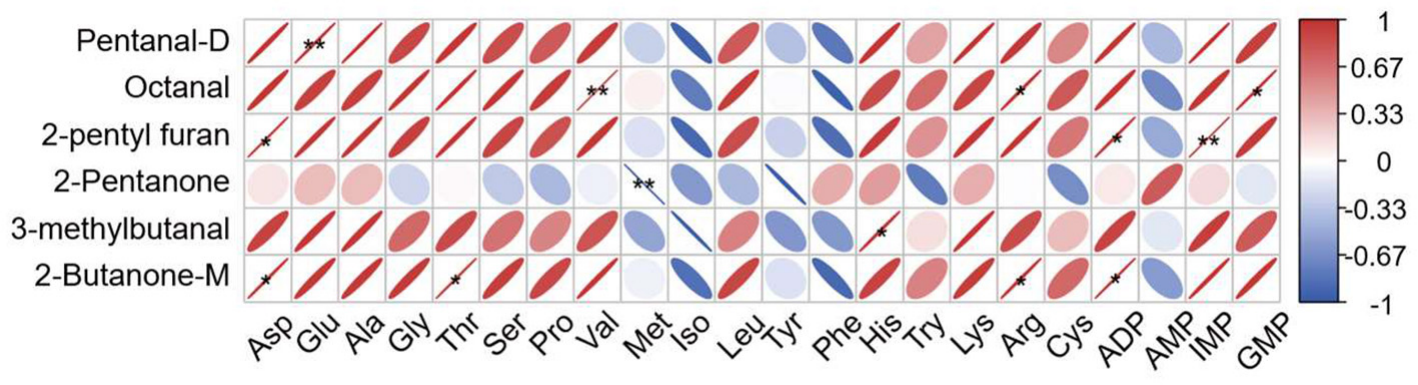
Correlation analysis of key volatile compounds and non-volatile compounds. Red represents positive correlation and blue represents negative correlation. *Indicates a statistically significant correlation (*P* < 0.05). **Indicates a statistically significant correlation (*P* < 0.01).

To further investigate the relationship between flavor compounds, Pearson correlation analysis was performed between key volatile compounds and the non-volatile taste substances in beef supplemented with dietary OEO. The analysis revealed several significant correlations (|*r*| > 0.70, *P* < 0.05), as shown in Figure 5. Aldehydes, a major group of key volatile compounds, showed pronounced positive correlations with several free amino acids and nucleotides. Notably, Pentanal exhibited a strong positive correlation with Glu. Octanal was significantly correlated with Val, Arg, and GMP. Meanwhile, 3-methylbutanal was positively correlated with His. Other Volatile Compounds, such as 2-butanone were strongly correlated with Asp, Thr, and Arg. 2-pentyl furan was positively correlated with Asp and IMP. In contrast, a significant negative correlation was observed between 2-Pentanone and Met.

## 4 Discussion

Proximate analysis serves as the direct method for assessing the basic nutritional composition of meat. Consistent with previous reports by Pukrop et al. (20) and Wang et al. (21), our study found that dietary supplementation with OEO did not significantly alter the proximate composition of beef, as evidenced by comparable moisture, crude fat, crude protein, and ash among the OEO and CON groups (*P* > 0.05). Although no statistical differences were detected, numerical differences between the CON and OEO groups were observed. Such numerical variations may influence subsequent changes in meat composition and could help explain the effects on flavor components and quality due to the intrinsic characteristics of these variables.

Amino acids are the primary taste-active compounds in meat and key precursors for the development of flavor characteristics via the Maillard reactions and lipid oxidation (22, 23). Among them, umami, an important flavor attribute of meat (24), is primarily linked to Glu and Asp. Glu also acts as a flavor enhancer and helps regulate acid-base balance in muscle tissues (25). Complementing the umami taste, sweet amino acids further modulate flavor complexity by reducing bitterness and improving overall palatability (26, 27). Increasing these flavor-related amino acids therefore enriches the sensory profile of meat. In this study, the systemic elevation of total free amino acids across OEO groups indicated their positive effect on the flavor characteristics of beef. Notably, the HOE group showed significantly higher levels of umami amino acids, sweet amino acids, and total free amino acids, suggesting that OEO supplementation may promotes the deposition of taste-active amino acids and thereby enhances flavor. This effect may be attributed to the strong antioxidant activity of phenolic compounds in OEO, such as thymol and carvacrol, which scavenge reactive oxygen species and chelate pro-oxidant metal ions (28). Post-mortem muscle tissue is vulnerable to oxidative stress, which can lead to the oxidative degradation of proteins or amino acids, such as sulfur-containing amino acids (29). Dietary natural antioxidants like OEO have been reported to reduce post-mortem protein oxidation and denaturation (30, 31). This protective effect may help preserve the integrity of flavor-related amino acids and improve their accumulation, thereby maintaining more substrate available for flavor formation. Our findings are consistent with previous studies showing that dietary thymol or carvacrol promotes protein metabolism and raises levels of flavor-related amino acid (e.g., cystine) in poultry (32). Similarly, Xu et al. (33) found that adding 800 mg/kg apple polyphenols increased both total amino acids and volatile flavor compounds in pork, supporting the view that dietary antioxidants can promote flavor precursor accumulation.

Generally, the intensity of umami in meat is primarily influenced by the synergistic interaction between specific flavor amino acids and nucleotides (24, 37). The 5′-nucleotides, including GMP, IMP, and AMP, are important of non-volatile flavor components. In post-mortem muscles, ATP is gradually hydrolyzed to ADP, then to AMP and IMP. IMP can be further converted into GMP (38, 39). These two nucleotides are known to act synergistically with Asp and Glu to enhance umami and sweetness (40, 41). In the present study, although dietary OEO supplementation did not lead to statistically significant increases in IMP or GMP levels, a numerical increase in both nucleotides was observed in the HOE group. Notably, ADP levels were significantly higher in the OEO-supplemented groups compared to the CON group. Given that ADP is an upstream metabolite in the metabolic pathway, its accumulation may partially explain the observed trend toward higher IMP and GMP concentrations. Similar observations have been reported in other studies. Li et al. (42) found that dietary supplementation with prickly ash seed, which is rich in flavonoids and phenolic compounds, increased ATP and its degradation products in lamb meat. Likewise, Shu et al. (43) reported that curcumin, another phenol-rich compound, elevated 5′-nucleotide levels in chicken meat and improved its flavor profile. These findings suggest that phenolic compounds may influence nucleotide metabolism, although the underlying mechanisms are not yet fully understood. Given the known synergistic umami-enhancing effects between 5′-nucleotide, Glu, and Asp, the higher levels of umami amino acids observed in the OEO group, together with the numerical increase in IMP, might collectively contribute to an improved umami taste (41). Nucleotide may also enhance umami through synergy with sweet amino acids (40). In conclusion, dietary OEO supplementation appears to promote the accumulation of certain flavor precursors, which may help enrich the taste profile of beef.

The aromatic profile of meat products is a main determinant of consumer preference, and volatile compounds regarded as core indicators for evaluating meat flavor quality. In the present study, combined electronic nose and GC-IMS analyses demonstrated that dietary supplementation with OEO significantly altered the volatile profiles of beef. Strong responses from sensors detecting nitrogen oxides, aromatic components, and organic sulfides were consistent with GC-IMS results showing significant differences in the volatile substances among the groups. Aldehydes were identified as the predominant volatile flavor components in beef in this study, mainly derived from lipid oxidation and amino acid degradation after post-mortem (34). The increased abundance of aldehydes in the OEO groups may be attributed to the phenolic active substances in OEO (such as carvacrol and thymol). These phenolic components possess potent antioxidant properties, which can inhibit excessive lipid peroxidation in meat (44, 45). In our previous research, the antioxidant regulation effect of OEO on lipid metabolism and oxidative stability in beef has been demonstrated, which can strongly support the regulation of OEO on lipid-derived flavor precursor substances in this study (46). Additionally, during post-slaughter aging, the abundant amino acids and nucleotides in muscle tissue can undergo mild spontaneous degradation reactions, providing precursor substrates for the generation of certain aldehydes, interacting with lipid-derived flavor compounds, further promoting an accumulation of aldehydes content. Similar regulatory mechanisms may also contribute to the changes in ketones and alcohols contents observed in this study. Our findings were consistent with previous reports on beef flavor characteristics (47), where aldehydes were also identified as the dominant volatile compounds. With the increase of dietary OEO level, the contents of most aldehydes showed an increase, leading to a significant increase in the total aldehyde content in beef. This trend was highly correlated with the observed changes in intramuscular fat and amino acids. The improvement of aldehyde content mediated by polyphenol-rich active compounds has also been reported in lamb and poultry meat studies, which further corroborates our results (48). Most saturated aldehydes (pentanal, hexanal, heptanal, nonanal) have low odor thresholds and strong odor activity, which play a decisive role in forming the basic flavor of meat (49). Hexanal was the most abundant volatile compound in all groups, contributing typical fruity and grassy aromas that form the core of beef flavor (50). In the aldehydes, pentanal, octanal, and 3-methylbutanal were identified as key flavor markers of beef. Pentanal is mainly derived from oleic acid oxidation (51), which contributes meat with fruity or berry-like flavors (52); Octanal can contribute similar fatty and fruity aroma characteristics (53, 54). In addition, 3-methylbutanal originates from the degradation of branched-chain amino acids, forming nutty and chocolate-like aromas (55, 56). These findings suggest that dietary OEO supplementation may optimize the volatile flavor of beef by increasing key aldehyde contents, thereby enriching the desirable fatty, fruity and nutty aromas of beef.

Ketones are an important category of volatile compounds in meat, typically contributing floral, fruity, and creamy characteristics. These compounds are mainly produced through lipid oxidation and amino acid degradation, thereby enriching the overall flavor profile of meat products (57). In this study, dietary OEO supplementation effectively increased the relative contents of most ketone compounds in beef. Specifically, the contents of 2-heptanone, 2-butanone, and 3-hydroxybutan-2-one were significantly higher in the HOE group than those in the CON group. As a key flavor-active compound, 2-butanone provides fruity and creamy notes to meat aroma (58), and 2-pentanone contributes a blue cheese characteristic (59). Together, these ketones cooperate to optimize the overall flavor of beef.

Alcohols, primarily generated from lipid oxidation (60), were the second most abundant volatiles identified in this study. Most alcohols present pleasant fatty, woody, and fruity aromas (61). Although most alcohols generally have high odor thresholds and exert limited flavor effects, certain long straight-chain alcohols, such as pentan-1-ol, has a relatively low thresholds (62) and impart distinct balsamic and fatty notes. In this study, pentan-1-ol was found to be higher in the OEO group, which is consistent with previous findings in yak meat flavor studies (63).

Furan derivatives are typical heterocyclic flavor substances closely related to unsaturated lipids oxidation, and 2-pentylfuran was detected as the representative compound in this study. Dietary OEO supplementation significantly elevated the relative content of 2-pentylfuran in beef. This compound is known to produce green and fatty notes (64), and was also identified a key marker compound distinguishing flavor differences between treatments in this study. Accordingly, the accumulation of 2-pentylfuran further enhanced the flavor characteristics, and coordinated with aldehydes, ketones and alcohols to jointly improve the flavor quality of beef.

Notably, the contents of esters including ethyl hexanoate, ethyl acetate, and methyl 2-propanoate were significantly decreased in OEO groups. Esters are mainly formed via the esterification between alcohols or acids, and commonly contribute a distinct wine-like aroma (65, 66). The two key factors that influence ester synthesis are the availability of precursor substrates and the activity of related enzymes (67). In this study, although volatile alcohol substrates were enriched in the OEO groups, OEO may mediate metabolic precursors toward the formation of other flavor substances, such as ketones (57). Furthermore, OEO may inhibited the esterification-related enzymatic activities involved in lipoxygenase driven cascade reactions (68), which could restrict the esterification between volatile alcohols and acids, ultimately contributing to the significant reduction in esters. Nevertheless, the specific regulatory mechanisms remain unclear, and targeted exploration is required in future research. Moreover, most esters generally exhibit a limited contribution to overall flavor due to their relatively high odor thresholds (69), which is consistent with our results showing no key volatile esters were identified in the OEO treatments. Consistent with previous studies, dietary nutrient regulation, particularly supplementation with natural plant extracts, can modulate the volatile flavor characteristics of meat (35, 43). In summary, OEO supplementation reduces the strong wine-like odors derived from esters, combined with the elevated fruity and floral notes provided by increased aldehydes, ketones, and alcohols, thereby helping form a more desirable flavor profile in beef.

The correlation analysis between volatile and non-volatile compounds further provides the potential effect by which dietary OEO regulates the beef flavor. Significant positive correlations were observed between key aldehydes and free amino acids as well as nucleotides in meat. Free amino acids such as Glu, Asp, Arg, and Thr are important non-volatile precursors that determine the meat taste (70). Key aldehydes, including pentanal, octanal, and 3-methylbutanal showed positive correlations with Glu, Arg, and His. This relation may indicated that the accumulation of these amino acids provided sufficient precursor substrates to favor the formation of flavor substance (56). Furthermore, ketones also presented correlation characteristics with amino acids. The positive correlation of 2-butanone with sweet amino acid (Thr) and umami amino acid (Asp) suggests a potential flavor synergy between tasting substances and volatile compounds (71, 72), which interactively enrich the taste and flavor. Notably, significant correlation of 2-Pentylfuran with umami-related Asp further enhance the link between amino acids degradation and flavor form. In summary, the integrated correlation analysis demonstrated that dietary OEO supplementation could alter the metabolic linkage between volatile aromatic compounds and non-volatile taste substances in beef. OEO could regulate the accumulation of flavor precursors such as free amino acids and nucleotides, further optimize the formation of volatile compounds, and ultimately improve the overall flavor quality and characteristics of beef.

## 5 Conclusion

This study demonstrated that dietary OEO supplementation contributes to a more desirable flavor profile in beef. HOE supplementation significantly increased the deposition of umami and sweet amino acids, as well as the content of nucleotide. Moreover, analysis of the volatile compounds revealed that HOE supplementation significantly increased the content of key volatiles, including 3-methylbutanal, pentanal, octanal, 2-butanone, 2-pentanone, and 2-pentyl furan, which collectively contribute to the flavor characteristics of beef. These findings indicated the role of OEO in promoting the accumulation of flavor precursors and the generation of volatile compounds, providing new insights into the application of natural plant additives for improving meat flavor quality. The present study primarily focused on the correlation between non-volatile and volatile flavor compounds. Future research can be conducted to clarify the molecular mechanism underlying the regulatory role of OEO in beef flavor formation.

## Data Availability

The raw data supporting the conclusions of this article will be made available by the authors, without undue reservation.
