# Supplementary material for: Non-volatile and volatile compound analyses revealed the effect of oregano essential oil on the flavor characteristics of beef
Source: Front Nutr. 2026 May 7;13:1832300. doi: 10.3389/fnut.2026.1832300 (PMC13190482; doi:10.3389/fnut.2026.1832300)
Supplement: Supplementary file 1 [file Table_1.docx]

Supplementary Table 1 Ingredients and nutrient composition of basal diets

| **Items** | **Experimental period (month)** | | | | | | | |
| --- | --- | --- | --- | --- | --- | --- | --- | --- |
|  | 1 | 2 | 3 | 4-7 | 8 | 9 | 10 | 11-13 |
| Ingredients (DM basis, %) |  | | | | | | | |
| Steam-flaked corn | 21.85 | 34.36 | 30.89 | 40.00 | 56.00 | 61.00 | 61.00 | 65.00 |
| Steam-flaked wheat | 4.00 | 2.00 | 2.00 | 5.59 | 2.00 | 2.00 | 2.00 | 2.00 |
| Steam-flaked barley | 4.00 | 2.00 | 2.00 | 3.61 | 2.00 | 2.00 | 2.00 | 2.00 |
| Steam-flaked black bean | 4.77 | 8.24 | 2.87 | 8.00 | 9.84 | 2.00 | 4.72 | 4.72 |
| Corm germ meal | 4.82 | 2.00 | 8.33 | 2.00 | 3.01 | 8.72 | 6.00 | 2.00 |
| Corn DDGS | 16.00 | 6.14 | 10.00 | 3.19 | 3.00 | 3.00 | 3.00 | 3.00 |
| NaHCO_3_ | 0.67 | 0.64 | 0.67 | 0.75 | 0.92 | 0.94 | 0.94 | 0.94 |
| NaCl | 0.67 | 0.64 | 0.67 | 0.75 | 0.92 | 0.94 | 0.94 | 0.94 |
| Limestone | 0.90 | 0.70 | 0.60 | 0.63 | 0.50 | 0.50 | 0.50 | 0.50 |
| Premix^1^ | 1.33 | 1.29 | 1.34 | 1.50 | 1.82 | 1.90 | 1.90 | 1.90 |
| Corn stalk silage | 41.00 | 42.00 | 40.62 | 34.00 | 20.00 | 17.00 | 17.00 | 17.00 |
| Nutrient levels, % |  | | | | | | | |
| Crude protein | 12.60 | 11.55 | 11.27 | 11.30 | 11.95 | 11.46 | 10.09 | 10.09 |
| ADF | 16.02 | 15.38 | 15.67 | 12.90 | 8.92 | 7.95 | 7.87 | 7.87 |
| NDF | 29.31 | 26.20 | 28.38 | 22.63 | 17.51 | 16.03 | 15.87 | 15.87 |
| TDN^2^, % | 72.15 | 72.26 | 72.00 | 73.99 | 77.35 | 78.07 | 77.87 | 77.87 |
| NEm^2^, Mca/100 kg | 174.17 | 177.48 | 175.93 | 185.08 | 200.77 | 204.54 | 204.98 | 204.98 |
| NEg^2^, Mcal/100 kg | 112.33 | 114.71 | 113.45 | 122.65 | 136.44 | 139.95 | 140.39 | 140.39 |

^1^ Contained (per kg): 900 IU of vitamin A, 150 IU of vitamin D, 25 IU of vitamin E, 10 mg of Cu, 80 mg of Fe, 20 mg of Mn, 40 mg of Zn, 1.0 mg of I, 0.6 mg Se.

^2^ TDN, NE_m,_ and NE_g_ were calculated values according to NRC.
